# The polyether ionophore salinomycin targets multiple cellular pathways to block proliferative vitreoretinopathy pathology

**DOI:** 10.1371/journal.pone.0222596

**Published:** 2019-09-17

**Authors:** Alison M. Heffer, Jacob Proaño, Elisa Roztocil, Richard P. Phipps, Steven E. Feldon, Krystel R. Huxlin, Patricia J. Sime, Richard T. Libby, Collynn F. Woeller, Ajay E. Kuriyan

**Affiliations:** 1 Flaum Eye Institute, University of Rochester, Rochester, NY, United States of America; 2 Department of Environmental Medicine, University of Rochester, Rochester, NY, United States of America; 3 Center for Visual Sciences, University of Rochester, Rochester, NY, United States of America; 4 Department of Medicine, University of Rochester, Rochester, NY, United States of America; Bascom Palmer Eye Institute, UNITED STATES

## Abstract

Proliferative vitreoretinopathy (PVR) is characterized by membranes that form in the vitreous cavity and on both surfaces of the retina, which results in the formation of tractional membranes that can cause retinal detachment and intrinsic fibrosis of the retina, leading to retina foreshortening. Currently, there are no pharmacologic therapies that are effective in inhibiting or preventing PVR formation. One of the key aspects of PVR pathogenesis is retinal pigment epithelial (RPE) cell epithelial mesenchymal transition (EMT). Here we show that the polyether ionophore compound salinomycin (SNC) effectively inhibits TGFβ-induced EMT of RPE cells. SNC blocks the activation of TGFβ-induced downstream targets alpha smooth muscle actin (αSMA) and collagen 1 (Col1A1). Additionally, SNC inhibits TGFβ-induced RPE cell migration and contraction. We show that SNC functions to inhibit RPE EMT by targeting both the pTAK1/p38 and Smad2 signaling pathways upon TGFβ stimulation. Additionally, SNC is able to inhibit αSMA and Col1A1 expression in RPE cells that have already undergone TGFβ-induced EMT. Together, these results suggest that SNC could be an effective therapeutic compound in both the prevention and treatment of PVR.

## Introduction

Proliferative vitreoretinopathy (PVR) is a condition that arises in 5–10% of rhegmatogenous retinal detachments (RDs) and is the leading cause of RD surgery failure [1]. PVR is characterized by pre-, sub-, or intra-retinal fibrosis (scarring) that can result in recurrent detachments [1,2]. PVR with recurrent retinal detachments requires additional surgical interventions and is associated with poor visual outcomes [3]. There are currently no treatments for PVR other than surgeries to remove the PVR membranes or excise portions of the retina. Pharmaceutical agents that inhibit PVR development during the retinal detachment repair process could potentially improve both the surgical success rates and visual outcomes.

PVR membranes are composed mainly of retinal pigment epithelial (RPE) cells, but also include glial cells, fibroblasts, and many types of immune cells [4–7]; none of which are normally found in the vitreous cavity. There is growing evidence that following a retinal break, a change in the physiological blood-retina barrier causes an influx of immune cells in the vitreous cavity, which produce a variety of cytokines and growth factors [8–11]. Retinal tears also lead to RPE cells being dispersed into the vitreous, where they become exposed to growth factors and cytokines [10,12,13]. In order for PVR membranes to form in the vitreous cavity and exert tractional forces, the RPE cells must not only survive and proliferate in this new environment, but also undergo epithelial-mesenchymal transition (EMT) into contractile fibrotic cells. Transforming growth factor-beta (TGFβ) is a key growth factor known to induce RPE cell EMT, and is present at high levels in PVR patients [14–16]. TGFβ is capable of driving RPE cell migration, promoting collagen gel contraction, and stimulating cell differentiation to fibroblasts and myofibroblasts [17–22]. Together, this supports the concept that TGFβ is a critical factor driving the formation of the tractional PVR membranes.

The polyether ionophore salinomycin (SNC) was previously identified in a small molecule screen as a candidate drug in the prevention of TGFβ-induced myofibroblast differentiation in fibroblasts [20]. In that study, it was shown that salinomycin acts by inhibiting TAK1/p38 signaling in the non-canonical TGFβ pathway, and indirectly through Smad2 signaling [20]. In order to investigate potential of SNC to inhibit or prevent PVR, the present study tested the hypothesis that SNC has the ability to inhibit TGFβ-induced RPE cell EMT, migration, and contraction. Furthermore, we studied the effect of SNC on RPE cells that have already undergone EMT.

## Results

### Salinomycin inhibits cell epithelial mesenchymal transition (EMT) in RPE cells

**Fig 1:**
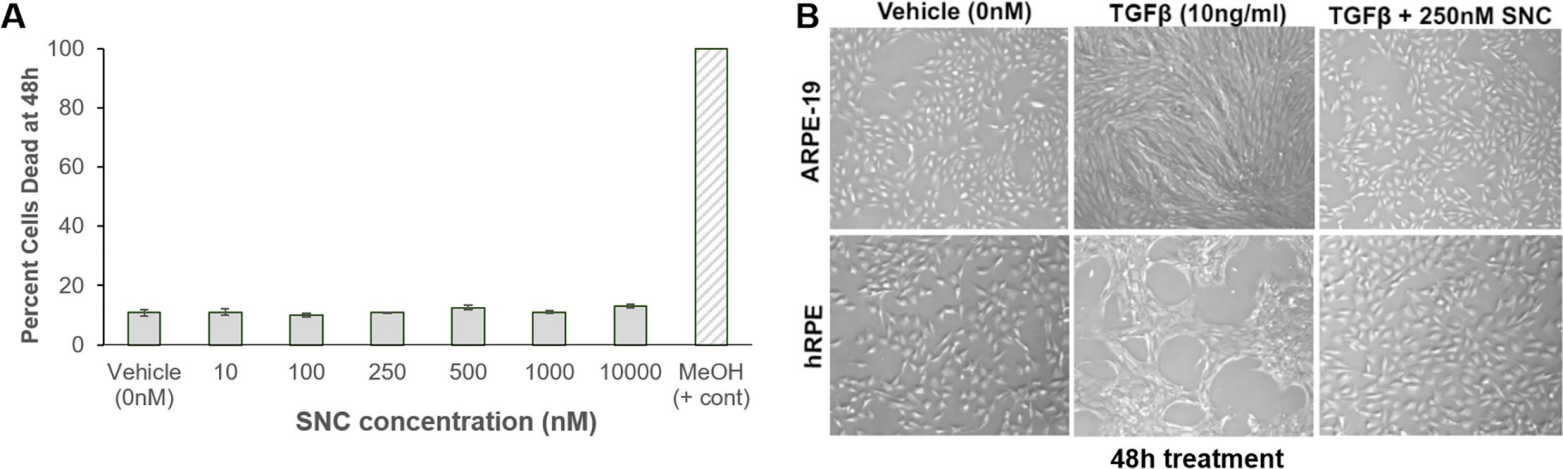
Salinomycin blocks RPE cell EMT with no toxicity. Cells were plated at a density of 10,000 cells/cm^2^ in media with 10% FBS for 24 hours, then starved in media with 0.1% FBS for 18 hours prior to vehicle/SNC treatment for 48 hours. A) Cytotoxicity, measured in ARPE-19 cells using a Live-Dead Assay after 48h treatment, showed no apparent increase in cell death over a wide range of SNC concentrations; 70% MeOH was used as a positive control for cell death. Each treatment was performed in triplicate and values were averaged together; similar trends in viability were seen with cells at different passages. B**)** Bright-field images of ARPE-19 and hRPE cells show that while TGFβ treatment promotes EMT to fibroblasts, 250nM SNC is sufficient to prevent RPE EMT to fibroblasts after 48h.

In order to study the ability of SNC to inhibit RPE cell EMT we used an *in vitro* cell culture model of PVR, in which the immortalized human ARPE-19 cell line and human primary cells (hRPE) were treated with TGFβ to induce EMT [23]. We simultaneously treated RPE cells with TGFβ and SNC for 48 hours to examine the ability of SNC to inhibit TGFβ-induced RPE EMT. SNC did not affect RPE cell viability at concentrations ranging from 10nM to 10μM (Fig 1A). Bright-field microscopy demonstrated that in both ARPE-19 and hRPE, addition of TGFβ resulted in a change in morphology of the cells into a spindle-shaped appearance that is consistent with EMT. This change was effectively inhibited with 250nM SNC treatment (Fig 1B), and not due to DMSO treatment (S1 Fig).

**Fig 2:**
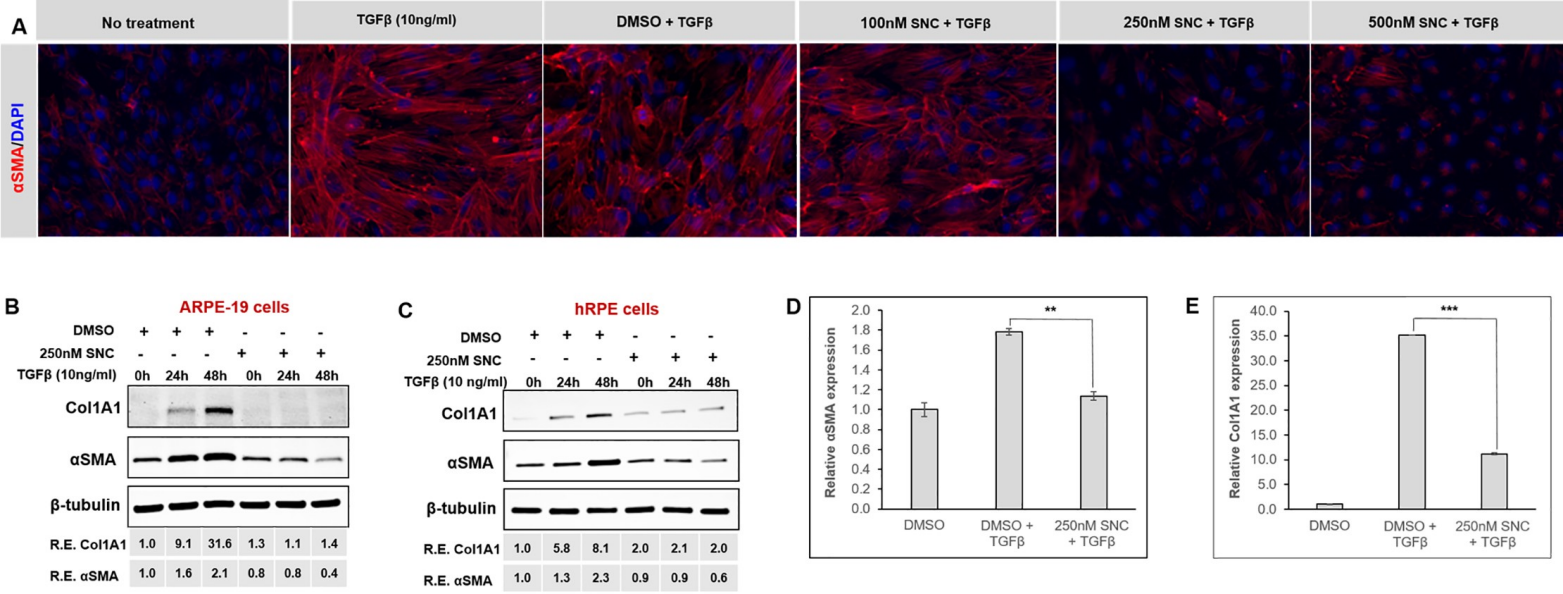
Salinomycin (SNC) treatment inhibits RPE TGFβ-induced EMT to fibroblasts. Cells were plated at a density of 10,000 cells/cm^2^ in media with 10% FBS for 24 hours, then starved in media with 0.1% FBS for 18 hours prior to vehicle/SNC treatment. A) αSMA immunofluorescence in ARPE-19 cells showed that SNC inhibits differentiation in a dose-dependent manner after 48h treatment. ARPE-19 (B) and hRPE (C) cells were stimulated with TGFβ and simultaneously treated with SNC or DMSO for up to 48 hours. Cells were collected and harvested immediately before treatment (0h) and after 24 and 48 hours of DMSO or SNC treatment. Expression of αSMA and Col1A1 were analyzed by western blotting. qPCR analysis in ARPE-19 cells shows up-regulation of αSMA (D) and Col1A1 (E) transcripts after 24h TGFβ stimulation; this upregulation was attenuated in the presence of SNC. Experiments were performed independently at least three times; similar trends in protein expression were reproducible in both RPE types at different passages. Representative western blots are shown. **p<0.001, ***p<0.0001, ANOVA with Tukey post-hoc analysis.

Immunofluorescence imaging was used to demonstrate that αSMA expression, a major marker of EMT, was strongly induced by TGFβ at 48 hours and was inhibited in a dose-dependent fashion by SNC (Fig 2A). Similarly, Western blotting analysis for αSMA as well as another marker of EMT, collagen 1 (Col1A1), demonstrated a 2-fold and 32-fold induction, respectively, after 48 hours of TGFβ treatment, which was markedly inhibited by 250nM SNC (Fig 2B and 2C). RT-qPCR analysis for αSMA and Col1A1 mRNA levels demonstrated a 1.8-fold and 35-fold induction, respectively, with 24 hours of TGFβ treatment in ARPE-19 cells. Treatment with 250nM SNC resulted in a 1.6-fold inhibition of αSMA (p<0.001) and 3-fold inhibition of Col1A1 after 24 hours (p<0.0001, Fig 2D). Taken together, these results show that SNC is effective at inhibiting TGFβ-induced RPE cell EMT without causing cell toxicity.

### Salinomycin inhibits TGFβ-induced cell migration and contraction in RPE cells

**Fig 3:**
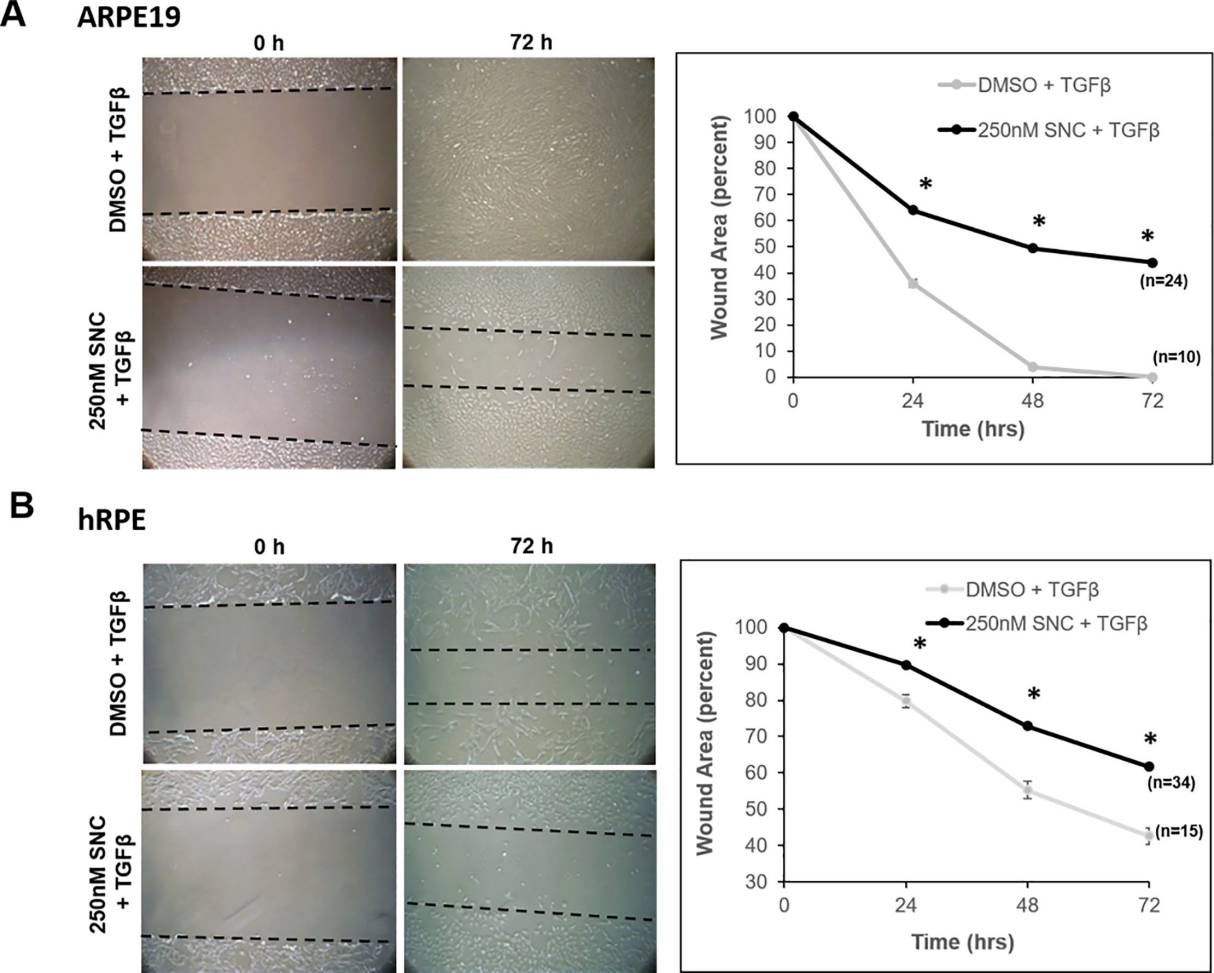
Salinomycin (SNC) inhibits TGFβ-induced cell migration in RPE cells. Two parallel scratches were introduced ~20mm apart in a well of confluent cells. Cell migration was stimulated with TGFβ and cells were simultaneously treated with DMSO or SNC for 72 hours. The same eight locations in each well were imaged immediately after treatment began (T = 0) and after 24, 48 and 72 hours of treatment. In both ARPE-19 cells (A) and human primary RPE cells (B) stimulated with TGFβ, 72 hours of SNC treatment inhibited cell migration to close the wounded area compared to vehicle (DMSO) treatment. Differences in migration between DMSO and SNC treatments were seen starting at 24 hours, and persisted at 72 hours, in both RPE lines. All wound areas were quantified using ImageJ. The initial wound area for each well was set at 100% and all subsequent time-points are shown as the percent of wound area remaining. The number of wound areas measured at each time point for the different treatment are listed next to each graphed line. *p<0.0000, ANOVA with Tukey post-hoc analysis.

To test whether or not SNC could inhibit migration of RPE cells, we performed a standard wound-healing assay, where a scratch was introduced into a confluent monolayer of RPE cells [24]. Cell migration was measured across the wound upon stimulation with TGFβ over 72 hours, with or without SNC treatment. Cells treated with TGFβ migrated to close the induced wound by 72 hours and treatment with SNC slowed cell migration and wound closure in both ARPE-19 and hRPE cells in a dose-dependent manner (Fig 3; S2 Fig, S3 Fig). In the ARPE-19 cell line, while all wound areas treated with TGFβ had filled by 72 hours, those treated with 250nM showed a 40–50% decrease in cell migration (p<0.0001, Fig 3A). A similar trend was seen in the primary RPE cells, though due to slower cell growth in general, none of the DMSO-treated wounds closed completely. Nevertheless, there was significantly more migration with DMSO-treated cells, compared to SNC-treated cells (p<0.0001, Fig 3B).

**Fig 4:**
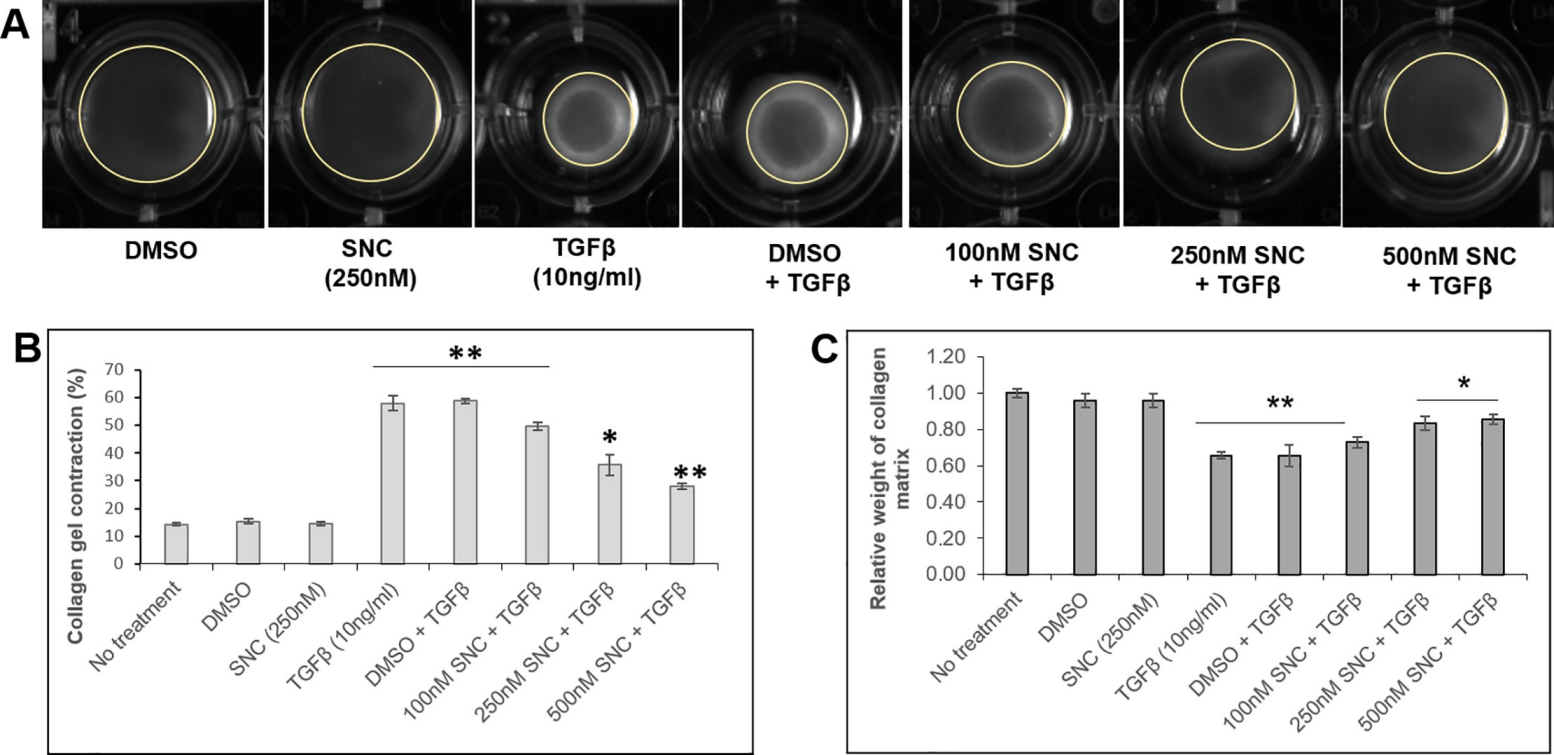
Salinomycin (SNC) inhibits TGFβ-induced contraction of a collagen matrix in ARPE-19 cells. A) ARPE-19 cells (2–3 x 10^5^ total cells) were embedded in a collagen matrix and incubated in media with the different treatments shown. After 72h of treatment, SNC+TGFβ, but not DMSO+TGFβ, inhibited the contraction of a collagen matrix in a dose-dependent manner. A representative gel area for each treatment is outlined with a yellow circle. Quantification of the areas measured (B) and weight of the collagen gel at 72 h (C) confirm that SNC inhibits contraction. Areas were quantified using ImageJ. *p<0.05; **p<0.01 (ANOVA with Tukey post-hoc analysis) when compared to untreated cells. All treatments were performed in triplicate and at different cell passages.

The fibrotic membranes that develop in PVR have contractile properties, which create a tractional force on the surface of the retina, resulting in recurrent retinal detachments in PVR patients [25,26]. We next examined whether SNC could inhibit TGFβ-induced contraction of a collagen matrix [27]. The contraction of the collagen matrix results in a lower weight of the collagen matrix [27]. Treatment with TGFβ (10ng/ml) resulted in ~60% contraction of the gel area after 72 hours (Fig 4A and 4B). Addition of 100, 250, and 500nM SNC produced a dose-dependent decrease in the percent contraction of the collagen matrix to 50%, 36% and 28% contraction, respectively (p<0.0001 for all doses, Fig 4A and 4B). The weight of the collagen gel was measured after 72 hours of SNC treatment to confirm that SNC had inhibited contraction of the collagen matrix (Fig 4C).

### Salinomycin treatment also targets differentiated myofibroblasts

**Fig 5:**
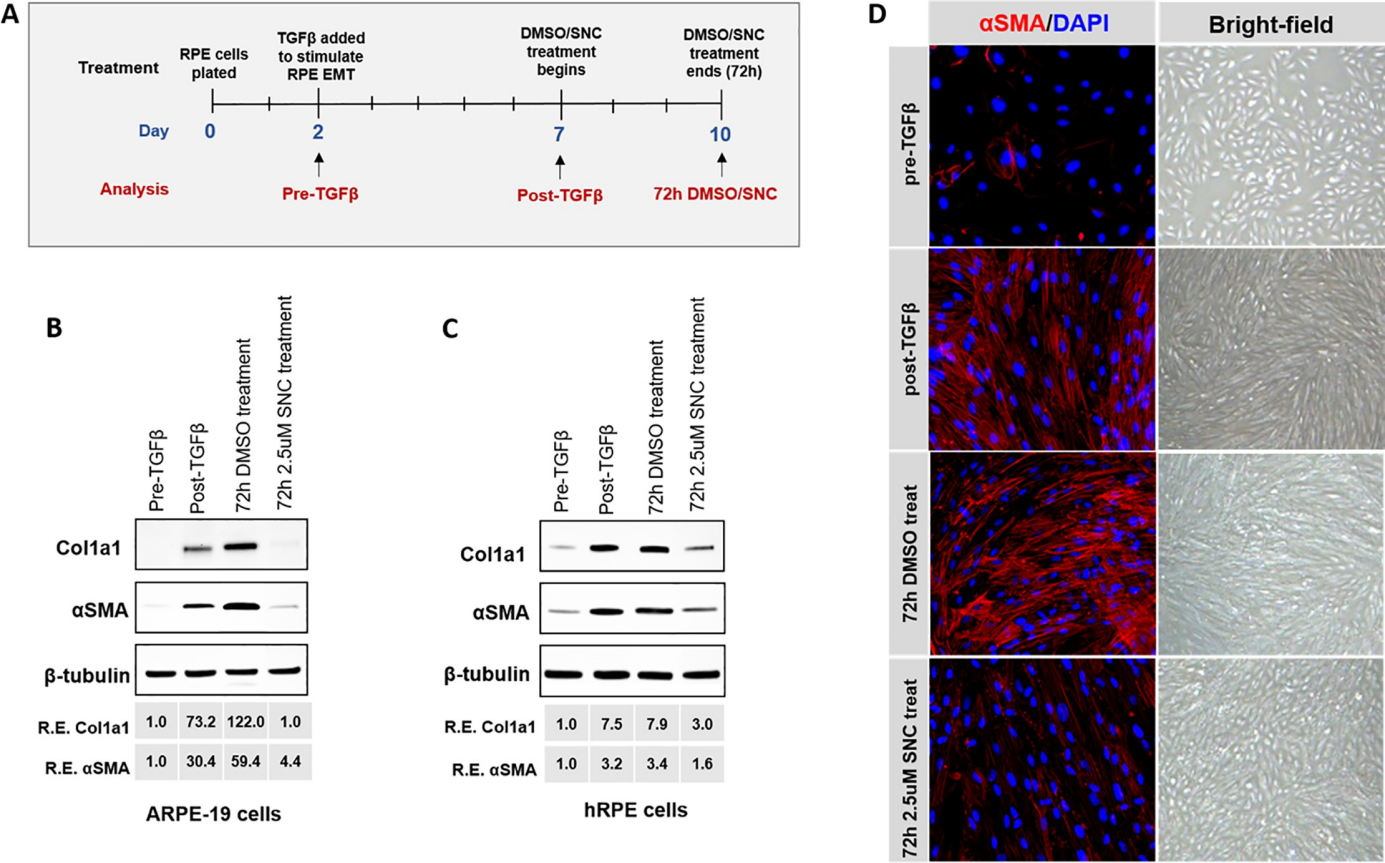
SNC inhibited expression of fibroblast markers in differentiated fibroblasts. A) A timeline showing collection and treatment points of RPE cells used in this experiment. ARPE-19 (B) and hRPE (C) cells were plated in media for 48 hours (“pre- TGFβ”) and then stimulated for 5 days with TGFβ (10ng/ml) to allow for cell differentiation to occur (“post- TGFβ”). Cells that had undergone TGFβ-induced EMT were then treated with media containing DMSO or SNC for an additional 72h and Col1A1 and αSMA protein levels were analyzed by western blotting. D) Immunofluorescence in ARPE-19 cells at the different analysis time-points confirmed the western blot data. Bright-field images also show that cells treated with SNC after EMT appear less fibrotic. All experiments were reproducible in both RPE cell lines at different cell passages. Immunofluorescence images were taken at 20X magnification and bright-field images at 4X magnification.

Since many PVR cases are not found or treated until the fibrotic tractional membranes have formed, we examined whether SNC treatment had any effects on RPE cells that have already undergone TGFβ-induced EMT (Fig 5). To study this, we stimulated RPE cells with TGFβ for 5 days, which as expected, promoted EMT as demonstrated by increased αSMA and Col1A1 expression (“post- TGFβ”; Fig 5B and 5C). Cells that had undergone TGFβ-induced EMT were then treated with dimethyl sulfoxide (DMSO, vehicle control) or 2.5μM SNC (with media containing 10% FBS to allow for cell survival) for 72 additional hours. After DMSO/SNC treatment, cells were harvested and the expression levels of αSMA and Col1A1 were analyzed (Fig 5B–5D). In ARPE-19 cells, TGFβ stimulated EMT, resulting in a 30- and 73- fold increase of αSMA and Col1A1, respectively (compare pre-TGFβ and post-TGFβ, Fig 5B and 5C). While there was an additional 2-fold increase in both of these EMT markers over the following 72 hours with DMSO treatment without SNC, SNC treatment markedly reduced αSMA and Col1A1 levels to near pre-TGFβ levels (Fig 5B; S4 Fig). We believe that this increase in EMT marker expression with DMSO treatment was due to the cells continuing to undergo EMT during these 72 hours of treatment and not DMSO promoting EMT, as DMSO treatment both alone and in the presence of TGFβ was not found to promote EMT in our cells (S1 Fig). In hRPE cells, a similar trend was found in αSMA and Col1A1 levels upon 5 days of TGFβ stimulation, though there was only a 3-fold increase in αSMA and 7.5-fold increase in Col1A1 (Fig 5C). Compared to DMSO treatment once the cells had undergone TGFβ-induced EMT, the addition of SNC reduced expression of both EMT markers by more than 2-fold (Fig 5C; S4 Fig). A reduction in αSMA expression was also shown by immunofluorescence in ARPE-19 cells treated with SNC (Fig 5D left column). Bright-field images of these cells show that SNC-treated cells appear to have less morphologic features of mesenchymal transition (fewer spindles and less flattened) than those treated with DMSO (Fig 5D, right column).

Additionally, we examined what effect SNC had on RPE cells embedded in a collagen matrix that had already undergone TGFβ-stimulated contraction for 72 hours. After collagen contraction occurred, matrices were weighed and transferred to solutions of media with 1% FBS, DMSO, or 2.5μM SNC for an additional 72 hours. We found that collagen matrices that were treated in 1% FBS-containing media or DMSO after initial collagen contraction showed a marginal increase in weight after 72 hours; those treated with 2.5μM SNC exhibited an additional ~25% increase in weight (p<0.01; S5 Fig). The increase in weight of the collagen matrix indicates a decrease in collagen contraction. Together, with the protein experiments above, these results show that in RPE cells that have already undergone TGFβ-induced EMT, SNC is effective at inhibiting expression of EMT markers and contraction.

### Salinomycin targets both canonical and non-canonical TGFβ signaling pathways

**Fig 6:**
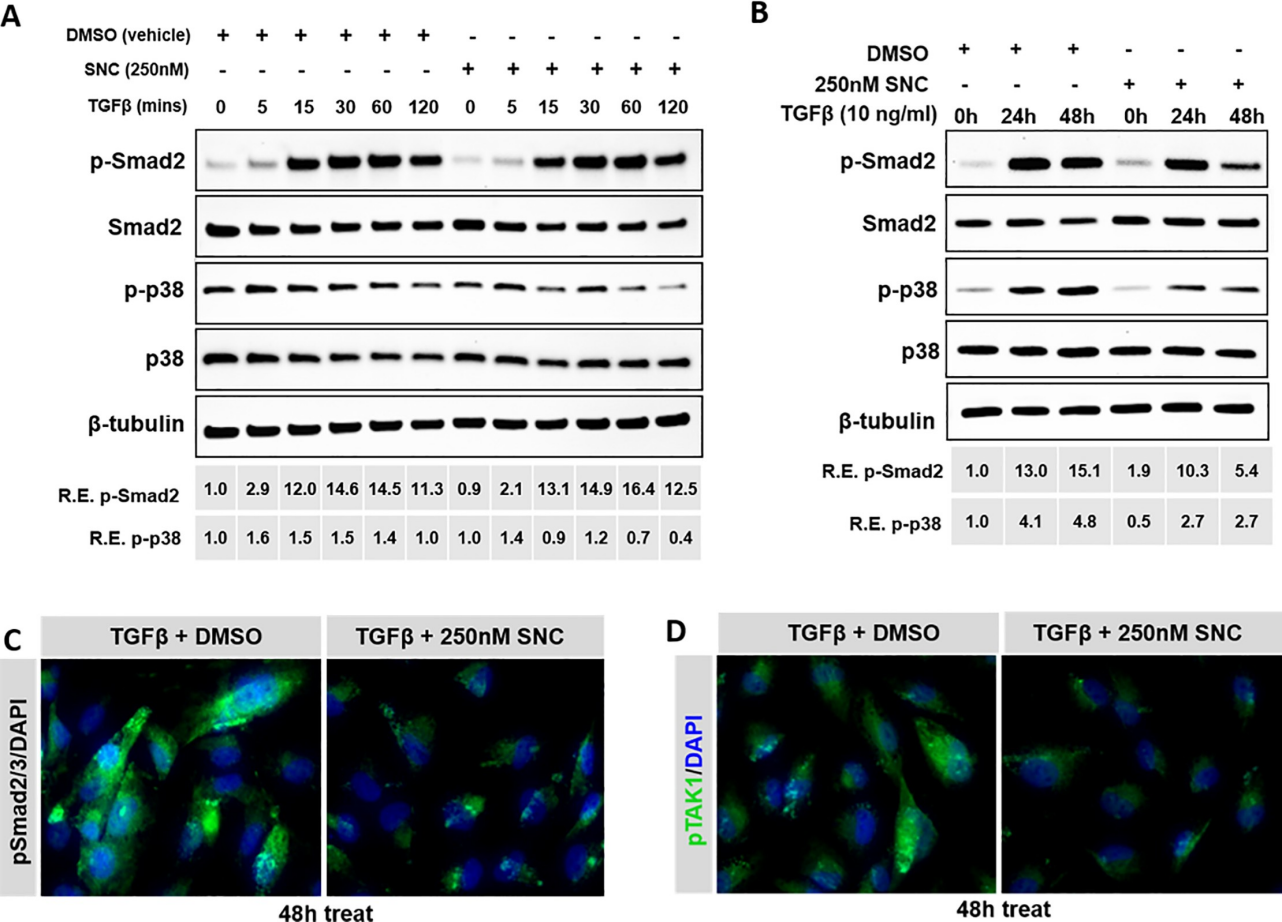
SNC targets both the p38 and pSmad2 pathways upon TGFβ stimulation. ARPE-19 cells were pre-treated with 250nM SNC for 1 hour before TGFβ was added. Western blotting of protein samples collected from early (A) and later (B) time-points of TGFβ stimulation were analyzed. We find that pSmad2 levels are not initially effected by SNC treatment, but p-p38 levels are lower. By 24 hours, we begin to see differences in pSmad2 levels, which persist at 48 hours, showing that SNC indirectly targets Smad2 signaling. Immunofluorescence staining of ARPE-19 cells after 48 hours of TGFβ/SNC show that both pSmad2/3 (C) and pTAK1 (D) expression is reduced.

In other studies involving fibroblasts, it has been shown that salinomycin acts by inhibiting TAK1/p38 signaling in the non-canonical TGFβ pathway, and indirectly through Smad2 signaling [20]. Additionally, others have reported that TGFβ rapidly stimulates TAK1/p38 signaling in ARPE-19 cells, which then indirectly activates Smad signaling, leading to EMT [28]. To build upon these findings, we examined whether or not SNC functioned via a similar mechanism in RPE cells. We found that shortly after TGFβ stimulation, cells treated with SNC showed a 1.5 to 2-fold decrease in phospho-p38 expression compared to cells treated with DMSO (Fig 6A and 6B), which persisted at later time-points as well (Fig 6C and 6D). No significant difference in phospho-Smad2 expression levels was seen within the first 24 hours of TGFβ/SNC exposure (Fig 6A and 6B), however, phospho-Smad2 expression levels were 3-fold lower than vehicle-treated cells after 48 hours of SNC treatment (Fig 6B and 6C). Additionally, in cells treated with TGFβ, the addition of SNC showed a decrease in immunofluorescence staining of both pTAK1 and pSmad2/3 compared to vehicle at 48 hours (Fig 6C and 6D).

**Fig 7:**
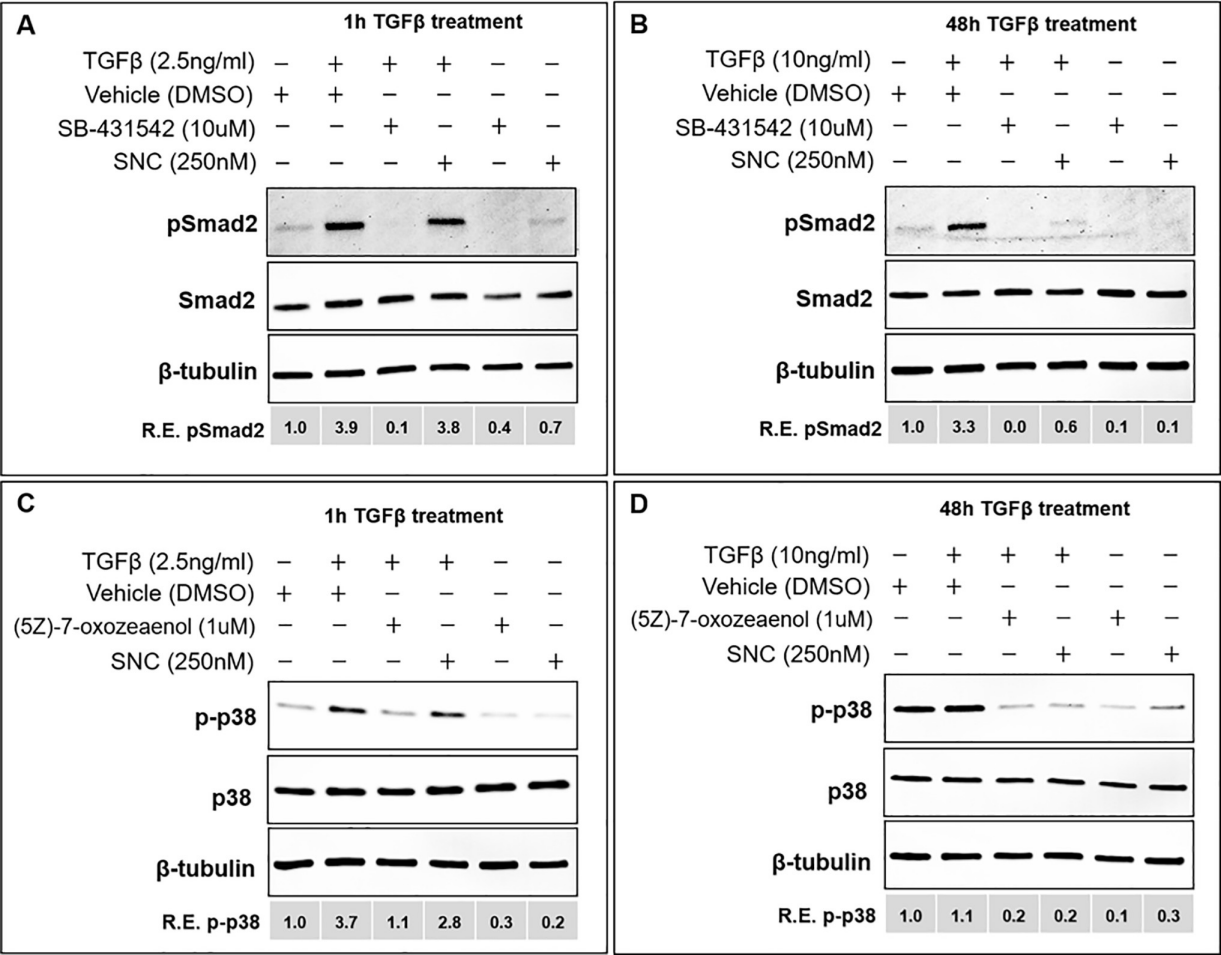
Inhibitors targeting canonical and non-canonical TGFβ signaling show that SNC first targets TAK/p38 signaling and then indirectly Smad signaling. Cells were plated at a density of 10,000 cells/cm^2^ in 10% FBS media for 24 hours, then starved in 0.1% FBS media for 18 hours. Cells were incubated in vehicle, SNC or inhibitor for 1 hour prior to TGFβ stimulation for 1 hour or 48 hours. A) Smad signaling was affected after 1h TGFβ stimulation in cells treated with the Smad inhibitor SB-431542 but not SNC. B) After 48 hours, cells treated with TGFβ in the presence of both SB-431542 and SNC showed decreased Smad signaling. C) TAK signaling was affected both after 1 hour (C) and 48 hours (D) in cells treated with either the TAK inhibitor (5Z)-7-oxozeaenol or SNC upon TGFβ stimulation. Experiments were performed independently at least two times; similar trends in protein expression were reproducible at different cell passages. Representative western blots are shown.

**Fig 8:**
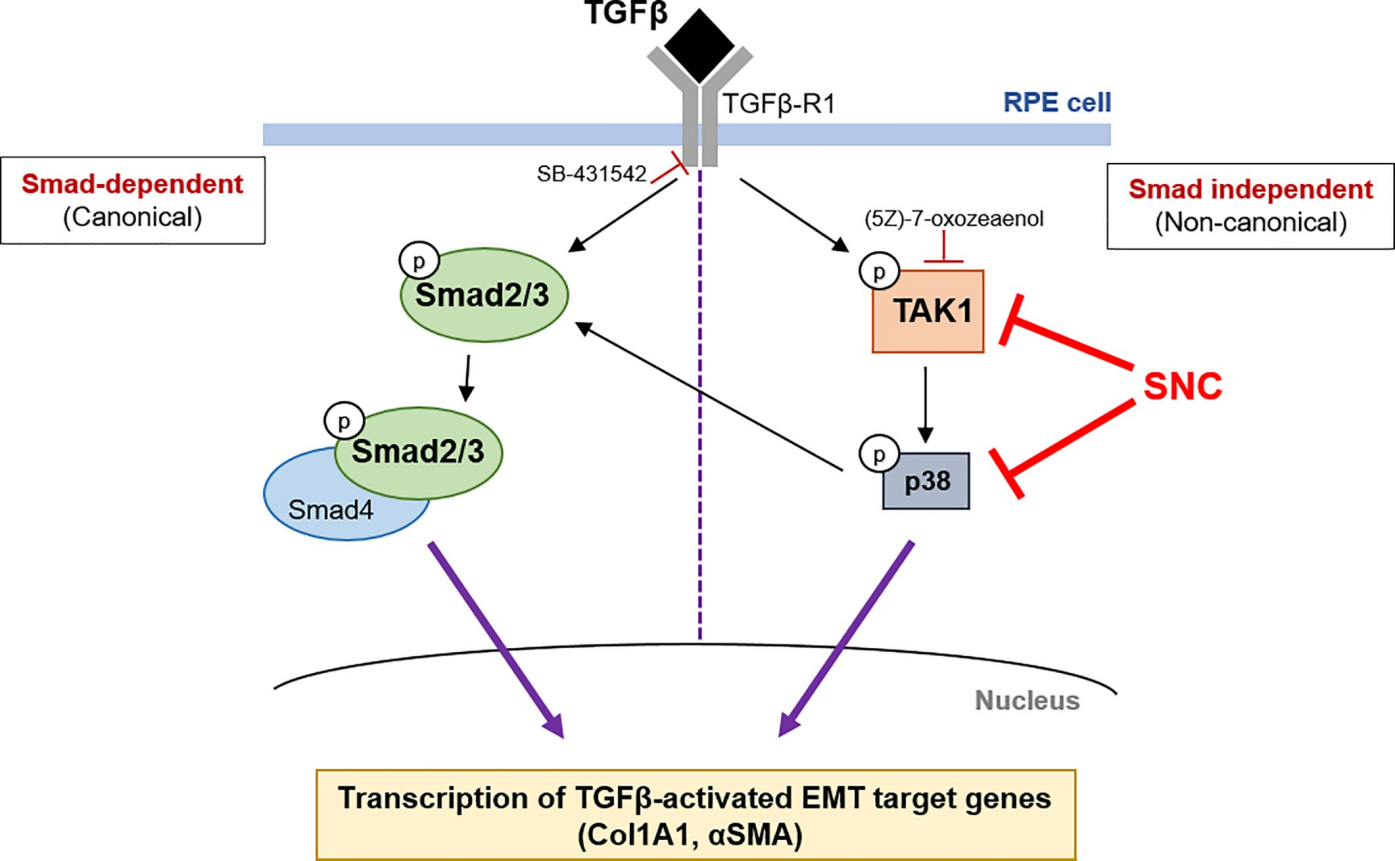
A model of salinomycin’s mechanism of action in RPE cells. Upon TGFβ binding to the TGFβ receptor, components of both Smad2/3 and TAK1 signaling pathways are rapidly phosphorylated and activated. TAK activation leads to the phosphorylation and activation of p38, which leads to a further increase in Smad2/3 phosphorylation and activation. Together, these signaling pathways lead to the transcription and activation of genes involved in EMT, including Col1A1 and αSMA. Salinomycin inhibits phosphorylation and downstream activities of TAK1 and p38, including the activation of Smad2/3 and ultimately EMT.

To further support that salinomycin attenuates EMT by targeting TAK1/p38 signaling early and Smad signaling later, we used (5Z)-7-oxozeaenol and SB431542 inhibitors to abolish TAK1/p38 and Smad signaling, respectively (Fig 7; [29,30]). Indeed, we find that while the Smad inhibitor completely blocked pSmad2 expression after 1 hour of TGFβ activation, SNC did not significantly affect the expression of pSmad2 at this time (Fig 7A). However, after 48 hours of TGFβ stimulation, both the Smad inhibitor and SNC had a similar effect on TGFβ-induced Smad signaling (Fig 7B). The TAK1 inhibitor reduced expression of TGFβ-induced phospho-p38 expression both at 1 hour and 48 hours (Fig 7C and 7D), showing that TAK1/p38 signaling is effected early and continues to be important in TGFβ signaling in RPE cells. While we also see an increase in phospho-p38 levels in vehicle-treated cells after 48 hours (Fig 7D), this is likely due to effects of stress and starvation documented in ARPE-19 cells in serum-free media [31]. Together, these results suggest that salinomycin first targets the TAK1 non-canonical TGFβ signaling pathway, and then indirectly, the canonical Smad2/3 TGFβ pathway, which is consistent with previous findings (Fig 8; 20).

Lastly, to examine whether both Smad and TAK signaling are required for TGFβ-induced EMT in RPE cells, we examined the expression of Col1A1 and αSMA in ARPE-19 cells treated with either the Smad inhibitor, TAK inhibitor or SNC for 48 hours (S6 Fig). We find that inhibition of either Smad signaling or TAK signaling resulted in a loss of RPE cell EMT, similar to that seen in cells treated with SNC. Combined with the results from Figs 6 and 7, we have shown that SNC inhibits TAK/p38 signaling with subsequent inhibition of Smad signaling and both of these pathways are important in TGFβ-induced EMT in RPE cells.

## Discussion

SNC, a polyether ionophore, was isolated from *Streptomyces albus* and has been used for over 30 years as an antibiotic for livestock to prevent coccidiosis and improve nutrient absorption and feed efficiency [32]. Recently, SNC has gained attention for its anti-cancer properties [33–35] and ability to suppress TGFβ-induced EMT in human breast cancer cells [36]. In addition, a recent study found that SNC has potent anti-scarring effects in fibroblasts [20]. SNC’s anti-scarring properties also make it an attractive potential agent for combating PVR, a fibrotic process which occurs after retinal detachment and can lead to irreversible vision loss.

In order to study the potential of SNC to inhibit the scarring process associated with PVR, we used an established *in vitro* RPE cell culture model of PVR, in which RPE cells plated at sub-confluent densities are exposed to TGFβ to promote EMT, as demonstrated by increased expression of early fibrotic markers [23]. RPE cells stimulated with TGFβ exhibit behaviors of cells involved in PVR, including migration and contraction. TGFβ is a major cytokine that drives fibrosis in PVR and is elevated in the vitreous of patients with PVR [14–16]. In this work, we show that SNC is also an effective compound against several key aspects of PVR pathogenesis, including RPE cell EMT (Fig 2), migration (Fig 3, S2 and S3 Figs), and contraction (Fig 4). The anti-fibrotic effects of SNC on RPE cells occurs at concentrations that are not toxic to RPE cells (Fig 1). To ensure our findings were not unique to the immortalized ARPE-19 cell line, several key experiments were replicated in human primary RPE cells. It is noteworthy that the concentration of SNC we find effective in inhibiting TGFβ-induced EMT in RPE cells is much lower than that used to target cancer cells in preliminary human studies [33]. Together, these results suggest that SNC is capable of preventing all key cellular processes involved in PVR formation.

In addition to inhibiting RPE EMT, we show–for the first time—that SNC is also capable of decreasing expression of EMT markers (Col1A1 and αSMA) and collagen contraction in RPE cells after they have undergone TGFβ-induced EMT (Fig 5, S5 Fig). SNC treatment resulted in a large increase in the weight of the gel compared to the untreated control and DMSO treatments, which demonstrates reversal of previous TGFβ-induced contraction of the collagen matrix with SNC (S5 Fig). Our findings of TGFβ-inhibition resulting in some degree of RPE cell EMT reversal is supported by a study by Shih and colleagues, which demonstrated that treatment of human fetal RPE cell, which underwent EMT secondary to multiple passages, with a TGFβ-inhibitor, A-83-01, restored the RPE cell phenotype to some degree, based on transcriptome profiles and morphology [37]. SNC’s potential ability to reverse RPE cell EMT makes it an especially exciting potential agent for patients with PVR as it provides the opportunity to treat patients who have already developed scarring, instead of merely the prevention of scarring.

To understand how salinomycin targets RPE and fibrotic cellular processes involved in PVR membrane formation, we examined pathways that are known to be activated by TGFβ stimulation. The canonical TGFβ signaling cascade is through the Smad pathway [38,39]. Woeller and colleagues [20] found that SNC inhibited early phosphorylation of p38 and TAK1 and late phosphorylation of Smad2, and Dvashi and colleagues have found that TAK1/p38 signaling is directly activated by TGFβ and Smad signaling indirectly affected in ARPE-19 cells [28]. This led to the hypothesis that salinomycin blocks the TAK1-p38 signaling pathway, which results in inhibition of TGFβ-induced Smad2-dependent signaling. Indeed, we also found that SNC has an early inhibition of TGFβ-induced p38 phosphorylation (as early as 15 minutes), followed by late inhibition of TGFβ-induced Smad2 phosphorylation (at 48 hours). The delayed effect on Smad2 phosphorylation suggests an indirect role on this pathway component. The use of Smad and TAK1 inhibitor molecules confirmed that TAK1/p38 signaling is affected early by SNC and Smad signaling later (Figs 7 and 8). Future studies will investigate p38 inhibition as a potential target to inhibit RPE cell EMT.

In conclusion, our results demonstrate that SNC is effective at targeting major pathogenic processes of PVR (RPE cell migration, EMT, and contraction) *in vitro*. Furthermore, SNC is a unique potential therapy for PVR because it potentially reverses RPE cell EMT at concentrations that are non-toxic to RPE cells. SNC appears to be promising potential agent for PVR, which is a blinding disease process with no pharmacologic therapies currently. Future studies will investigate the ability of SNC to inhibit PVR in, pre-clinical animal models.

## Materials and methods

### Cell culture and treatment

Human ARPE-19 cells (ATCC, Manassas VA) were grown in HEPES-buffered DMEM and Ham’s F12 (1:1) supplemented with 10% fetal bovine serum (FBS; HyClone) and 1% anti-anti (Life Technologies) at 37°C/5% CO_2_. Cells (between passages 3 and 18) were plated at a density of 10,000 cells per well and grown in DMEM/F12 + 10% FBS for 24 hours, then starved in DMEM/F12 + 0.1% FBS for 16–18 hours before treatment to remove any response to TGFβ found in FBS. Salinomycin (Sigma, S4526) dissolved in DMSO was added to media with 0.1% FBS and then cells were assayed after 48 hours, unless otherwise indicated. SB-431542 (Sigma) and (5Z)-7-oxozeaenol (Tocris) were dissolved in DMSO and used at concentrations of 10uM and 1uM, respectively. Human primary RPE cells (Sciencell) were grown in EpiCM media supplemented with 2% FBS; starved cells were treated in EpiCM with no FBS added. Cells were plated and treated the same as ARPE-19 cells.

### Cell viability experiments

Cell viability was measured using the Live/Dead Cytotoxicity Assay (Molecular Probes). For this assay, both MeOH (shown in Results) and puromycin were to induce death in ARPE-19 cells; similar trends were observed with both. All experiments were done in triplicate and repeated 2 times; similar trends were seen between experiments.

### Wound-healing scratch assay

Cells were grown in 6-well plates until ~75% confluent, and then the media was changed to starved media for ~18 hours to ensure that any response the RPE cells may have to growth factors in serum media was minimized. Two parallel scratches, about 20mm apart, were introduced with a pipette tip in each well. The media and cell debris from the scratch were removed and media with TGFβ (Peprotech, Rocky Hill, NJ;10ng/ml), TGFβ + SNC or TGFβ + DMSO (vehicle control) was added. The same eight locations in each well were imaged immediate after the treatment began (T = 0) and after 24, 48 and 72 hours of treatment. By 72 hours, the scratch was completely filled in the TGFβ-treated samples, so no later time points were needed. The wound area of the identical images over time was measured in ImageJ (NIH) and percent wound healing was determined by setting the wound area at T = 0 to 100%, as described previously [20].

### Collagen contraction assays

The Collagen Contraction Kit (Cell Biolabs) was used for all assays, following the manufacturer’s protocol. Briefly, ARPE-19 cells (2–3 x 10^5^ cells) and 500μl collagen suspension were mixed and plated in a 24-well plate. The collagen gel was incubated at 37°C until it polymerized (~2 hours). For experiments which examined the effect of SNC on TGFβ-induced contraction, SNC/DMSO + TGFβ (10ng/ml) was added in 1ml media to the desired concentration and gels were carefully detached from the well. The gel was imaged at T = 0 and after an appropriate treatment time, and gel areas were measured in ImageJ. Percent contraction was measured in each well by setting the area at T = 0 to 100% and subtracting the percentage area that remained at 72 hours. The weights of all collagen gels were measured on an analytical balance. All treatments were performed in triplicate with the gel areas and weights averaged. For experiments that examined the effect of SNC on a collagen matrix that had undergone contraction, ARPE-19 cells were embedded in a collagen matrix as described above, and incubated in media containing TGFβ (10ng/ml) for 72h. Collagen matrices were weighed and then placed in a new well containing media with 1% FBS, DMSO or 2.5uM SNC for 72h. Collagen matrices were weighed again and compared to their original weight after TGFβ-induced contraction.

### Western blotting

At the desired time-point, media was removed and cells were washed once in PBS. Total protein was isolated by lysing the cells in 60mM Tris-HCl (pH 6.8) with 2% SDS containing 1X protease inhibitor mixture (Sigma). Lysates were sonicated for 5 seconds to shear genomic DNA. Protein concentrations were measured using a detergent-compatible protein assay (Bio-Rad). Total protein (5–10μg) was separated on a 4–20% TGX gradient gel (Bio-Rad) and transferred to a PVDF membrane (Millipore). The following antibodies were used to detect protein products at the expected size: Col1A1 (goat, 1:200, Santa Cruz), αSMA (mouse, 1:6000, Sigma), β-tubulin (rabbit, 1:1000, Cell Signaling), phospho-Smad2 (rabbit, 1:1000, Cell Signaling), Smad2 (rabbit, 1:1000, Cell Signaling), phospho-p38 (rabbit, 1:1000, Cell Signaling), p38 (rabbit, 1:1000, Cell Signaling). Appropriate HRP-conjugated secondary antibodies (1:5000, Jackson ImmunoResearch) were used and detected with Western-Lightening Plus-ECL (Perkin Elmer). Band intensities were quantified using ImageLab software (Bio-Rad). Protein levels in each lane were normalized to β-tubulin.

### Immunofluorescence

ARPE-19 cells were fixed in 24-well plates at the desired time-point in 4% PFA for 20 minutes at room temperature. Cells were washed twice in PBS, permeabilized with 0.3% TritonX for 30 minutes, washed several times in PBS/0.01% TritonX/0.1% Tween20, and blocked for 1 hour in Blocking Solution (3% BSA, 1% serum, 0.01% TritonX, 0.1% Tween20, 300mM glycine). Cells were incubated in primary antibody overnight at 4°C in Blocking Solution without glycine. After several washes, cells were incubated in a fluorescence-conjugated secondary antibody. Cells were incubated in DAPI (1:1000, Invitrogen Molecular Probes) for 1 hour before imaging. All imaging was done on the ZOE imaging system (Bio-Rad). Antibodies used for immunofluorescence were: αSMA-Alexa-Fluor555 (mouse, 1:1000, Abcam), phospho-Smad2/3 (rabbit, 1:200, Cell Signaling), phospho-TAK1 (Thr187) (rabbit, 1:200, Bioss), Alexa Fluor-488 secondary (goat, 1:500, Invitrogen Molecular Probes).

### RNA isolation and RT-qPCR analysis

Total RNA was isolated from cells using the TRIzol reagent and manufacturer’s protocol (Invitrogen). RNA quality and concentration were measured using a NanoDrop 1000. cDNA was synthesized using the QuantiTect Reverse Transcription Kit (Qiagen) and 25ng was used as a template in each reaction. Primers were designed to amplify a region spanning an exon-exon border to avoid possible background from any genomic DNA contamination. GAPDH was used as a control. Primer sequences used were: *GAPDH*: 5’-AATCCCATCACCATCTTCCAG-3’ and 5’- ATGACCCTTTTGGCTCCC-3’; *αSMA*: 5’-TGCAGAAAGAGATCACCGC-3’ and 5'-CCGATCCACACCGAGTATTTG-3’; *Col1A1*: 5'-CCCCTGGAAAGAATGGAGATG-3’ and 5'- TCCAAACCACTGAAACCTCTG-3’. qPCR was performed using SsoAdvanced Universal SYBR Green Supermix and protocol (Bio-Rad). Each primer pair was run in triplicate on each cDNA sample. All calculations for relative expression levels were done using the comparative CT method described by [40]. Expression levels of all genes were normalized to GAPDH and expression in the DMSO (vehicle) control was set to 1.0.

### Statistical analyses

All data areas were analyzed using ImageJ (NIH). For determining statistical significance, one-way ANOVA tests followed by Tukey’s post-hoc tests were done. All p-values < 0.05 were considered significant and are given in the figure legends.

## Supporting information

S1 Fig DMSO alone does not promote EMT in RPE cells. A) ARPE-19 cells treated with DMSO or DMSO+TGFβ (10ng/ml) for 48 hours did not show a difference in expression of EMT markers compared to cells treated with media or media with only TGFβ (10ng/ml). B) Treatment with increasing concentrations of DMSO showed no effect on expression of EMT marker αSMA after 48 hours. The maximum DMSO concentration tested was the maximum amount used as a vehicle control in other experiments. Experiments were repeated at least twice independently and representative blots are shown.
(TIF)

S2 Fig Salinomycin (SNC) inhibits TGFβ-induced cell migration in ARPE-19 cells in a dose-dependent manner. A) Higher SNC concentrations show less migration of cells across the wound area. B) Quantification of the wound area over 72 hours with different SNC treatments. Differences in migration between DMSO and SNC treatments are seen starting at 24 hours in both RPE lines; all p-values are statistically significant (*p<0.0000, ANOVA with Tukey post-hoc analysis). All wound areas were quantified using ImageJ. The initial wound area for each well was set at 100% and all subsequent time-points are shown as the percent of wound area remaining.
(TIF)

S3 Fig Salinomycin (SNC) inhibits TGFβ-induced cell migration in human primary RPE (hRPE) cells in a dose-dependent manner. A) Higher SNC concentrations show less migration of cells across the wound area. B) Quantification of the wound area over 72 hours with different SNC treatments. Differences in migration between DMSO and SNC treatments are seen starting at 24 hours in both RPE lines; all p-values are statistically significant (*p<0.0000, ANOVA with Tukey post-hoc analysis). All wound areas were quantified using ImageJ. The initial wound area for each well was set at 100% and all subsequent time-points are shown as the percent of wound area remaining.
(TIF)

S4 Fig Quantification of SNC inhibition of fibroblast marker expression in differentiated fibroblasts. Relative expression of Col1A1 and αSMA in ARPE-19 (left column) and hRPE cells (right column) at the four time-points where cells were harvested and protein levels analyzed (see Fig 5). All experiments were repeated at least three times, with reproducible trends in both RPE cell lines at different cell passages. Averages of protein levels from each experiment are shown. Statistical analyses were performed between cells that had undergone TGFβ-induced EMT followed by 72h DMSO treatment vs 72h SNC treatment. ***p<0.001, **p<0.01, *p<0.05 (ANOVA with Tukey post-hoc analysis).
(TIF)

S5 Fig Salinomycin targets RPE cells in a collagen matrix. ARPE-19 cells were treated with TGFβ (10ng/ml) for 72h. Weights of the contracted collagen matrices were measured (post-TGFβ contraction) and then the gels were transferred to 1% FBS-containing media, DMSO, or SNC for 72h. All collagen matrices were then weighed again. After 72h in media containing SNC, collagen matrices increased ~25% weight compared to controls, which increased ~5% from pre-treatment weight. **: p<0.01 compared to both controls ANOVA with Tukey post-hoc analysis.
(TIF)

S6 Fig Inhibition of either TAK/p38 or Smad signaling is sufficient to prevent EMT. Cells were pre-treated with SB-431542, (5Z)-7-oxozeaenol or SNC for 1hour before TGFβ was added for an additional 48 hours. Analysis of EMT markers Col1A1 and αSMA by western blotting shows that cells treated with either inhibitor did not show an increase in EMT, similar to that seen with SNC. Experiments were repeated at least twice independently and representative blots are shown.
(TIF)
