# Supplementary figures and images for: The polyether ionophore salinomycin targets multiple cellular pathways to block proliferative vitreoretinopathy pathology
Source: PLoS One. 2019 Sep 17;14(9):e0222596. doi: 10.1371/journal.pone.0222596 (PMC6748436; doi:10.1371/journal.pone.0222596)

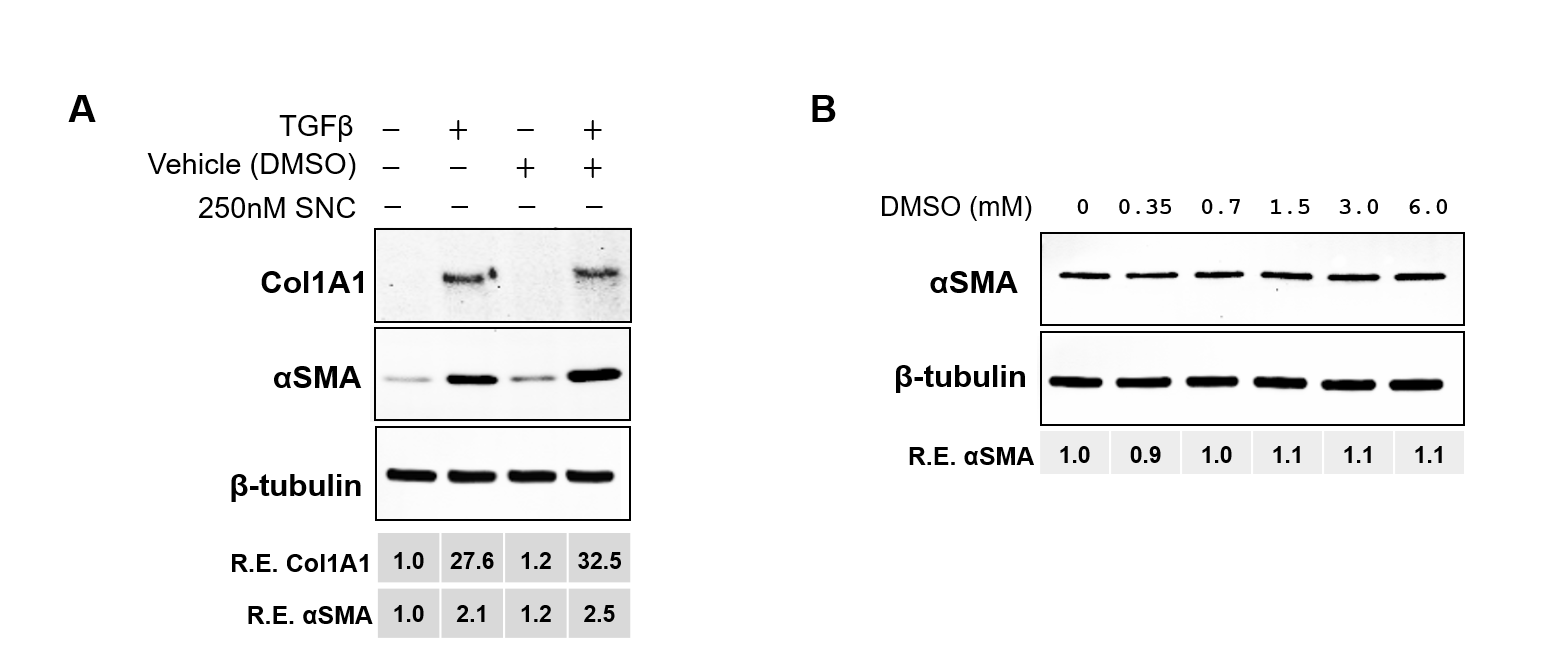

Supplement: S1 Fig — A) ARPE-19 cells treated with DMSO or DMSO+TGFβ (10ng/ml) for 48 hours did not show a difference in expression of EMT markers compared to cells treated with media or media with only TGFβ (10ng/ml). B) Treatment with increasing concentrations of DMSO showed no effect on expression of EMT marker αSMA after 48 hours. The maximum DMSO concentration tested was the maximum amount used as a vehicle control in other experiments. Experiments were repeated at least twice independently and representative blots are shown. (TIF) [file pone.0222596.s001.tif]

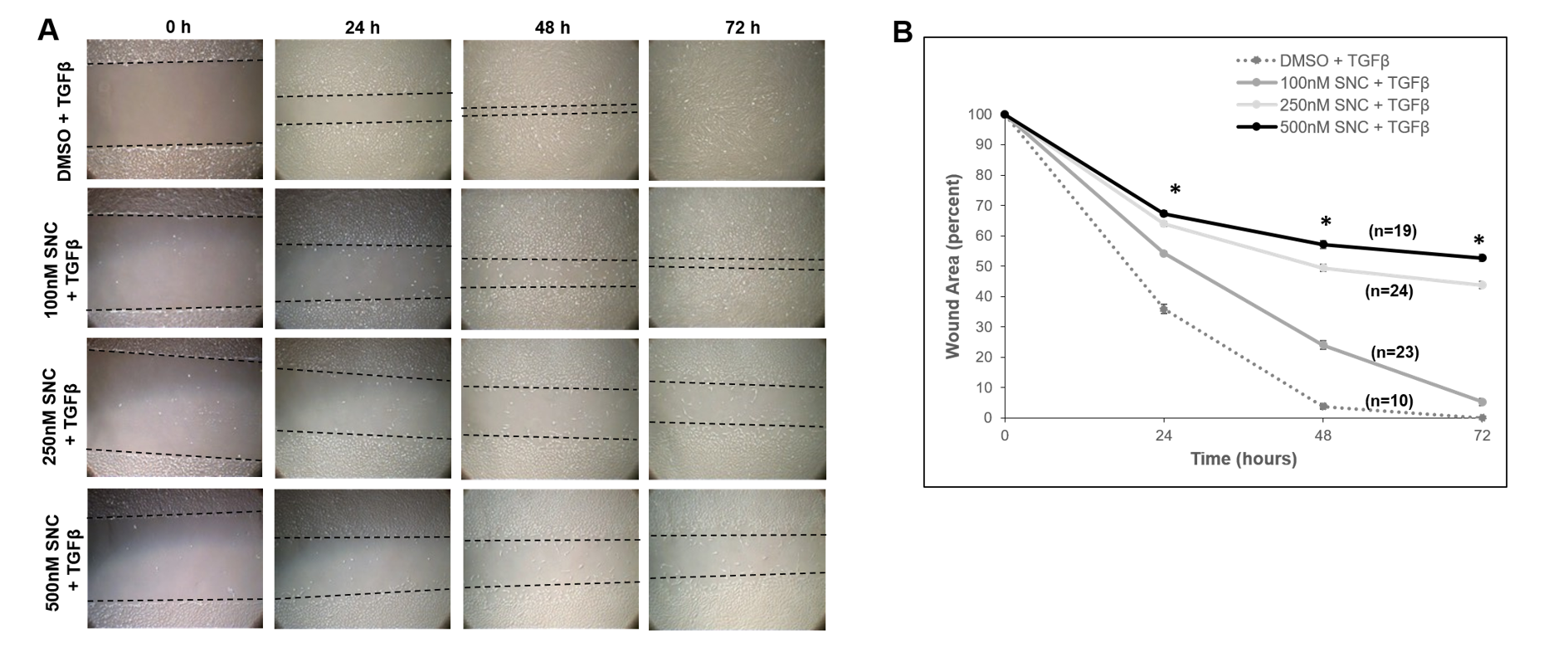

Supplement: S2 Fig — A) Higher SNC concentrations show less migration of cells across the wound area. B) Quantification of the wound area over 72 hours with different SNC treatments. Differences in migration between DMSO and SNC treatments are seen starting at 24 hours in both RPE lines; all p-values are statistically significant (*p<0.0000, ANOVA with Tukey post-hoc analysis). All wound areas were quantified using ImageJ. The initial wound area for each well was set at 100% and all subsequent time-points are shown as the percent of wound area remaining. (TIF) [file pone.0222596.s002.tif]

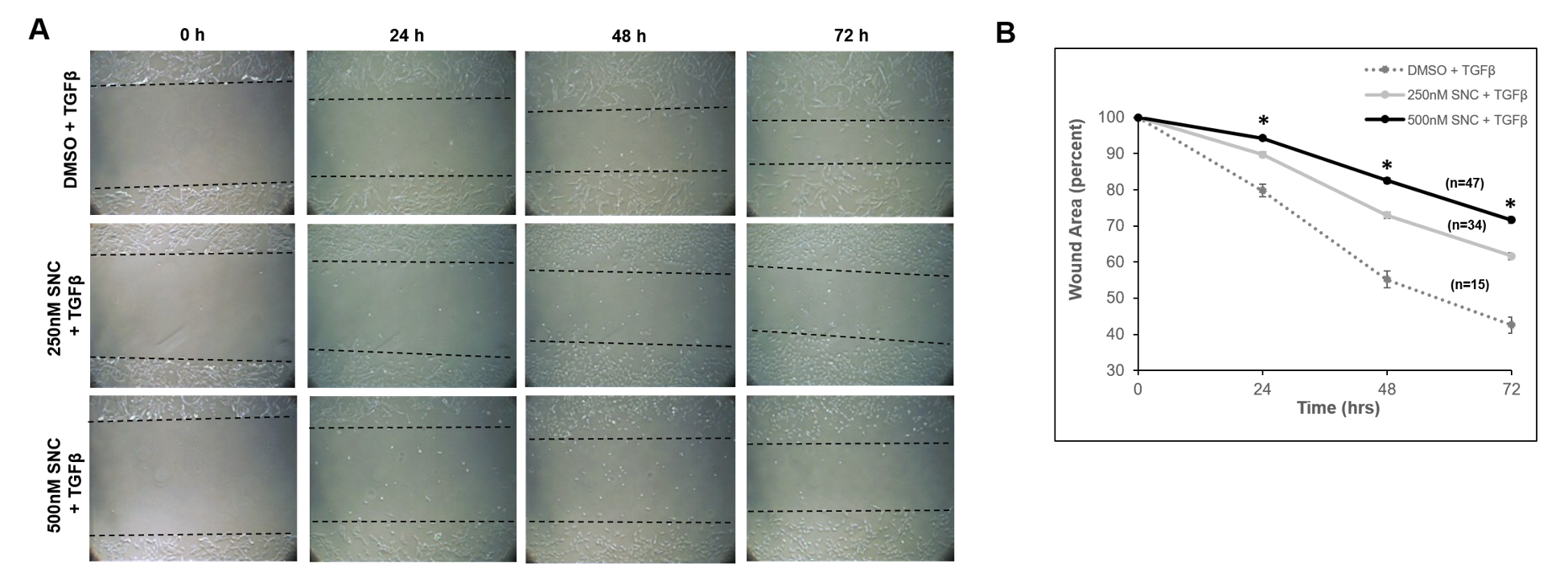

Supplement: S3 Fig — A) Higher SNC concentrations show less migration of cells across the wound area. B) Quantification of the wound area over 72 hours with different SNC treatments. Differences in migration between DMSO and SNC treatments are seen starting at 24 hours in both RPE lines; all p-values are statistically significant (*p<0.0000, ANOVA with Tukey post-hoc analysis). All wound areas were quantified using ImageJ. The initial wound area for each well was set at 100% and all subsequent time-points are shown as the percent of wound area remaining. (TIF) [file pone.0222596.s003.tif]

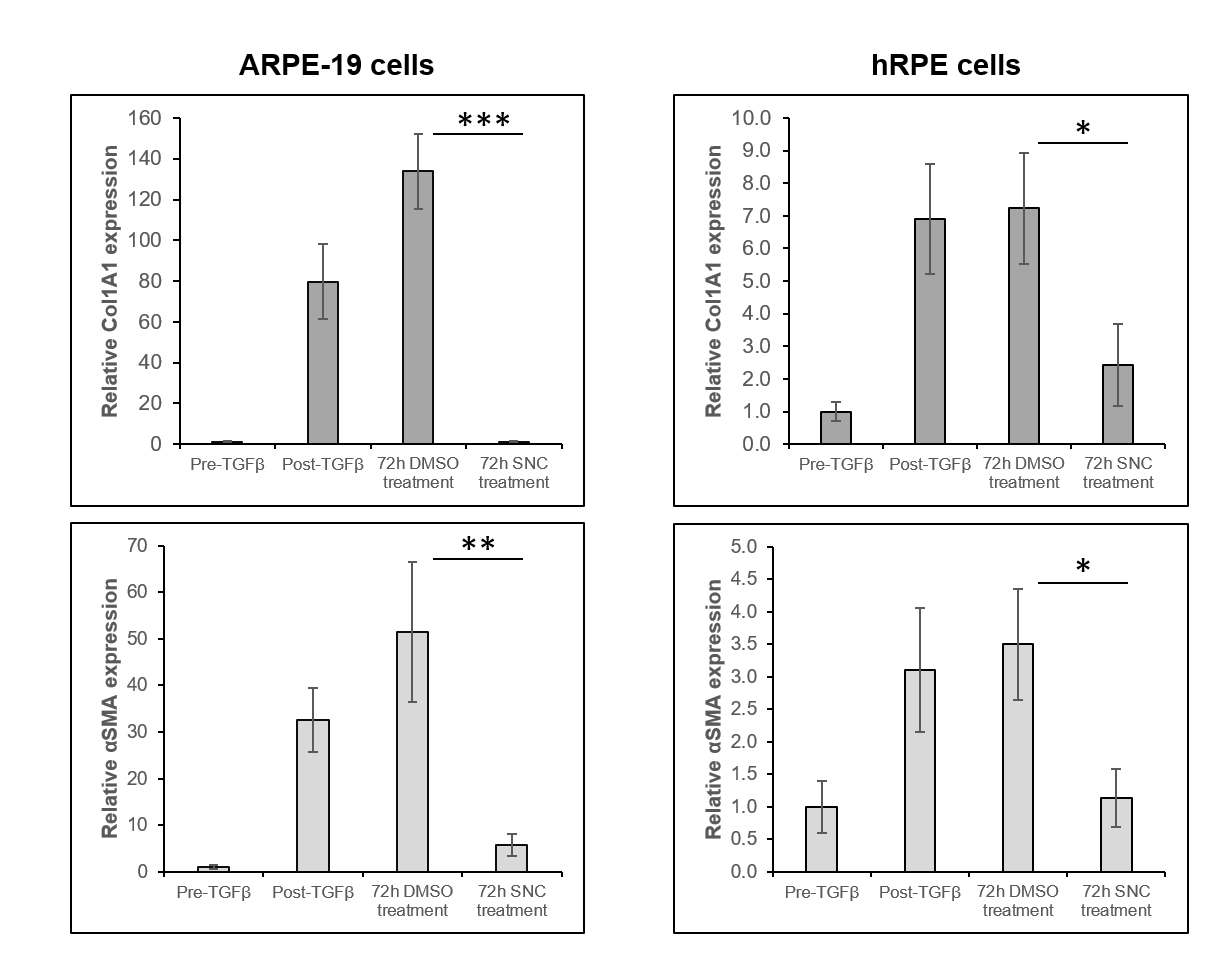

Supplement: S4 Fig — Relative expression of Col1A1 and αSMA in ARPE-19 (left column) and hRPE cells (right column) at the four time-points where cells were harvested and protein levels analyzed (see Fig 5). All experiments were repeated at least three times, with reproducible trends in both RPE cell lines at different cell passages. Averages of protein levels from each experiment are shown. Statistical analyses were performed between cells that had undergone TGFβ-induced EMT followed by 72h DMSO treatment vs 72h SNC treatment. ***p<0.001, **p<0.01, *p<0.05 (ANOVA with Tukey post-hoc analysis). (TIF) [file pone.0222596.s004.tif]

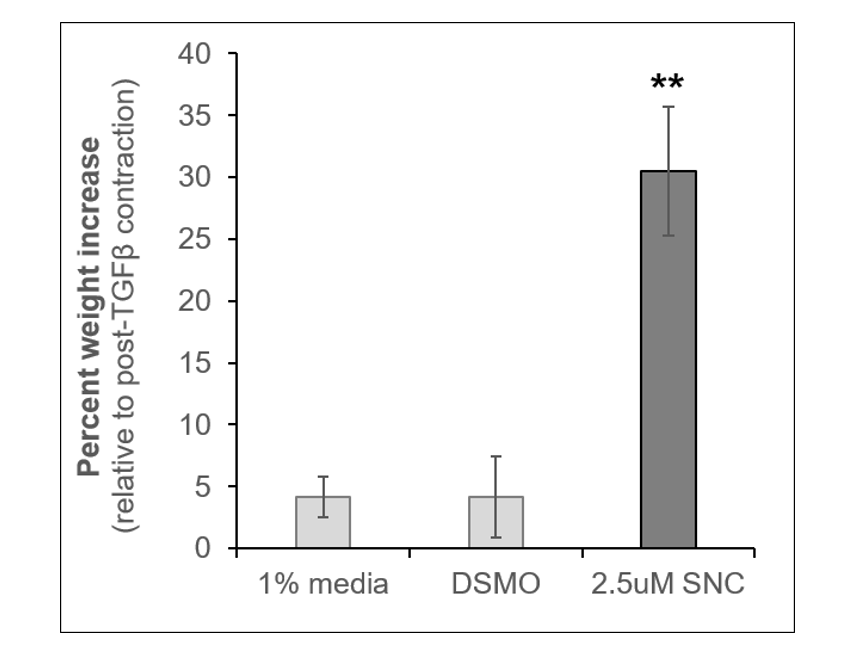

Supplement: S5 Fig — ARPE-19 cells were treated with TGFβ (10ng/ml) for 72h. Weights of the contracted collagen matrices were measured (post-TGFβ contraction) and then the gels were transferred to 1% FBS-containing media, DMSO, or SNC for 72h. All collagen matrices were then weighed again. After 72h in media containing SNC, collagen matrices increased ~25% weight compared to controls, which increased ~5% from pre-treatment weight. **: p<0.01 compared to both controls ANOVA with Tukey post-hoc analysis. (TIF) [file pone.0222596.s005.tif]

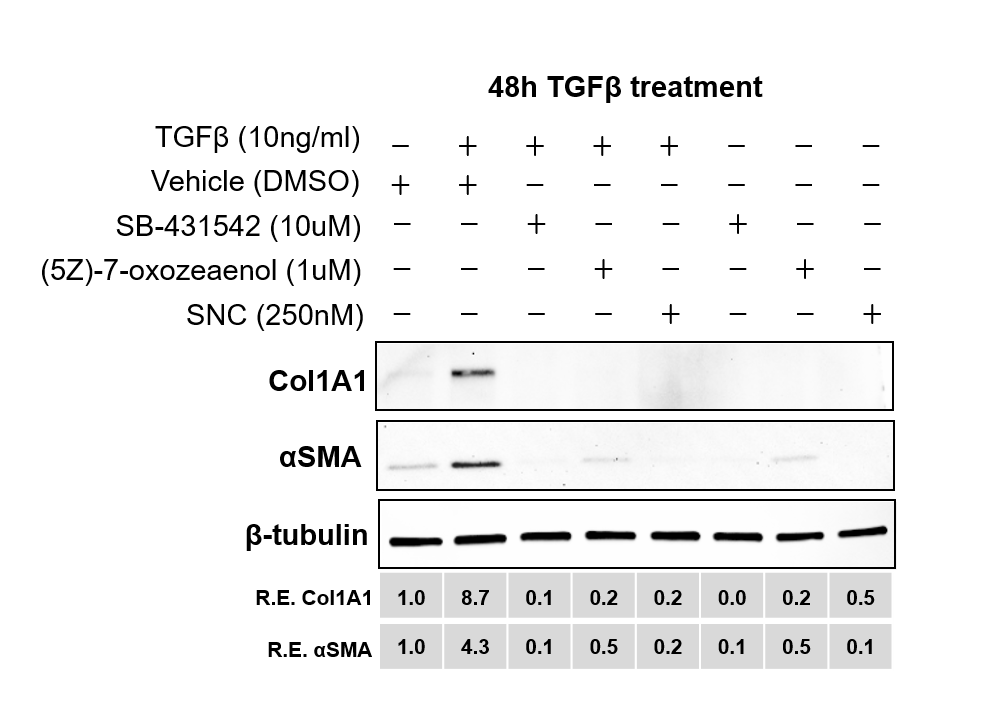

Supplement: S6 Fig — Cells were pre-treated with SB-431542, (5Z)-7-oxozeaenol or SNC for 1hour before TGFβ was added for an additional 48 hours. Analysis of EMT markers Col1A1 and αSMA by western blotting shows that cells treated with either inhibitor did not show an increase in EMT, similar to that seen with SNC. Experiments were repeated at least twice independently and representative blots are shown. (TIF) [file pone.0222596.s006.tif]
